# Molecular surveillance of the *Pfmdr1* N86Y allele among Congolese pregnant women with asymptomatic malaria

**DOI:** 10.1186/s12936-020-03246-0

**Published:** 2020-05-08

**Authors:** Louis Regis Dossou-Yovo, Francine Ntoumi, Felix Koukouikila-Koussounda, Jeannhey Christevy Vouvoungui, Ayodele Adedoja, David Nderu, Thirumalaisamy P. Velavan, Arsène Lenga

**Affiliations:** 1 Ecole Normale Supérieure, Marien Ngouabi University, Brazzaville, Republic of Congo; 2 Congolese Foundation for Medical Research, Brazzaville, Republic of Congo; 3 Faculty of Science and Technology, Marien Ngouabi University, Brazzaville, Republic of Congo; 4 School of Health Sciences, Kirinyaga University, Kerugoya, Kenya; 5 Institute of Tropical Medicine, University of Tübingen, Tübingen, Germany; 6 Vietnamese-German Center for Medical Research (VG-CARE), Hanoi, Vietnam; 7 Faculty of Medicine, Duy Tan University, Da Nang, Vietnam

**Keywords:** *Plasmodium falciparum*, *Pfmdr1*, Antimalarial drug resistance, Lumefantrine, Brazzaville, Republic of Congo

## Abstract

**Background:**

Malaria in pregnancy is associated with considerable morbidity and mortality. Regular surveillance of artemisinin-based combination therapy tolerance, or molecular makers of resistance, is vital for effective malaria treatment, control and eradication programmes. *Plasmodium falciparum* multiple drug resistance-1 gene (*Pfmdr1*) N86Y mutation is associated with reduced susceptibility to lumefantrine. This study assessed the prevalence of *Pfmdr1* N86Y in Brazzaville, Republic of Congo.

**Methods:**

A total 1001 of *P. falciparum*-infected blood samples obtained from asymptomatic malaria pregnant women having a normal child delivery at the Madibou Integrated Health Centre were analysed. *Pfmdr1* N86Y genotyping was conducted using PCR-restriction fragment length polymorphism.

**Results:**

The wild type *Pfmdr1* N86 allele was predominant (> 68%) in this study, whereas a few isolates carrying the either the mutant allele (*Pfmdr1* 86Y) alone or both alleles (mixed genotype). The dominance of the wildtype allele (*pfmdr1* N86) indicates the plausible decline *P. falciparum* susceptibility to lumefantrine.

**Conclusion:**

This study gives an update on the prevalence of *Pfmdr1* N86Y alleles in Brazzaville, Republic of Congo. It also raises concern on the imminent emergence of resistance against artemether–lumefantrine in this setting. This study underscores the importance to regular artemether–lumefantrine efficacy monitoring to inform the malaria control programme of the Republic of Congo.

## Background

*Plasmodium falciparum* malaria among pregnant women is a major public health concern in sub-Saharan Africa. Pregnant women have substantial risks and malaria in pregnant women are related to preterm delivery, intrauterine growth restriction, low birth weight and maternal anaemia. The World Health Organization (WHO) recommends the use of intermittent preventive treatment (IPTp) with sulfadoxine-pyrimethamine (SP) for pregnant women and also for infants (IPTi) [1]. Currently, artemisinin-based combination therapy (ACT) is the first-line treatment for *P. falciparum* uncomplicated malaria. Resistance against chloroquine (CQ) and its successor, sulfadoxine-pyrimethamine, had devasting consequences in sub-Saharan Africa in 1990s and 2000, particularly among children below 5 years [2].

After the introduction of ACT, malaria mortality and morbidity has globally declined until 2015. Even though ACT is still efficacious, there is sensitive concern on the potential spread of artemisinin-resistant *P. falciparum* parasites from Southeast Asia to sub-Saharan Africa, reminiscent of the spread of CQ and SP resistance [3–7]. The WHO recommends routine surveillance of anti-malarial drug efficacy once every 2 years. However, the efforts to monitor the emergence and spread of anti-malarial drug resistance in resource-limited settings are hampered due to high clinical trial costs. Molecular surveillance of distinct point mutation(s) in *P. falciparum* genes linked to anti-malarial treatment failure offers a cost-effective tool to monitor spatial and temporal emergence and spread of resistant parasites. High prevalence of gene mutations associated with CQ (*P. falciparum* chloroquine transporter, *Pfcrt*) and SP (*P. falciparum* dihydrofolate reductase gene; *Pfdhfr,* and *P. falciparum* dihydropteroate synthase; *Pfdhps*) resistance informed, in part, the decision to replace these anti-malarial drugs with ACT, including in the Republic of Congo [8–11].

The *P. falciparum multidrug resistance 1* protein *(PfMDR1),* also known as P-glycoprotein homologue, is a transmembrane protein of the *P. falciparum* digestive vacuole (DV) [12]. It is involved in the transport of substrates into digestive vacuole of the parasite, including anti-malarial drugs [13]. Distinct changes in the sequence and/or amplification of the copy number of the *Pfmdr1* gene alters *P. falciparum* susceptibility to several anti-malarial drugs [14].

In particular, the *Pfmdr1* N86Y single nucleotide polymorphism (SNP) has been implicated in *P. falciparum* resistance to chloroquine and amodiaquine [15]. *Pfmdr1* N86Y is mostly abundant in African settings. High prevalence of *Pfmdr1* 86 N and 86Y alleles is currently being driven by ACT-linked *P. falciparum* selection. Previous studies have shown that parasites carrying *Pfmdr1* N86 are less susceptible to lumefantrine [16, 17], artemether-lumefantrine (AL) selects for *Pfmdr1* 86 N, whereas artesunate-amodiaquine (ASAQ) and piperaquine is selective for *Pfmdr1* 86Y [16, 18–20]. Since this phenomenon indicates potential decline of malaria parasite sensitivity or increased tolerance to ACT partner drugs, *Pfmdr1* N86Y genotyping has been proposed as a useful marker to guide rotation of ACT medicines in a given geographical area [20, 21].

The present study aimed to genotype and to determine the prevalence of *Pfmdr1* N86Y in Brazzaville, Republic of Congo among pregnant women using maternal peripheral, placental, and cord blood. The study aims to provide factual data as a useful measure for the refinement and adaption of the current malaria treatment policy with the long-term goal of reducing malaria in the Republic of Congo.

## Methods

### Sample collection

This study analysed a total of 101 matched blood samples (maternal peripheral, placenta, and cord blood) collected from pregnant women with asymptomatic malaria who had a normal child delivery at the Madibou Integrated Health Center, Brazzaville, between March 2014 and April 2015 [21]. The study was conducted in Brazzaville, the capital of the Republic of Congo with 1.8 million inhabitants [22]. Malaria transmission in this area is perennial with *P. falciparum* being the predominant *Plasmodium* species [22, 23]. AL and ASAQ are the first-line and second-line anti-malarial drugs for uncomplicated *P. falciparum* malaria in the Republic of Congo, respectively [24].

### *Pfmdr1* genotyping

Total genomic DNA was isolated using QIAamp DNA Mini Kit (Qiagen, Hilden, Germany) according to the manufacturer’s instructions. Amplification of *P. falciparum* merozoite surface protein 2 gene (*Pfmsp2*) was used to determine *P. falciparum* multiplicity of infection (MOI), as described previously [25]. Nested-PCR followed by a restriction fragment length polymorphism (PCR–RFLP) were used to genotype *Pfmd1* N86Y, as described earlier [26]. In brief, *Pfmdr1* primary and nested PCRs were amplified by adding 2 µl DNA template into a PCR master mix (50 µl) containing 1X PCR buffer, 2.8 mM MgCl_2_, 200 µM dNTPs, 5 pM of each primer, 1UTaq DNA polymerases (Qiagen, Hilden, Germany). The primer pairs for the primary PCR were A1 (5′-CGGAGTGACCAAATCTGGGA-3′) and A3 (5′-GGGAATCTGGTGGTAACAGC-3′) and for the secondary PCR were A2 (5′-TTGAAGAACAGAAATTACATGATGA-3′) and A4 (5′-AAAGATGGTAACCTCAGTATCAAAGAAGAG-3′). The thermal cycler conditions were as follows: initial denaturation at 94 °C for 2 min, followed by 40 cycles at 94 °C for 1 min, 45 °C for 1 min, 72 °C for 1 min and a final extension at 72 °C for 5 min. The secondary reaction was amplified using the product of the primary reaction as a template. DNA extracted *P. falciparum* laboratory strains (3D7 and Dd2) and PCR grade water were used as positive and negative controls, respectively.

The *Pfmdr1* N86Y mutation was identified by digesting *Pfmdr1* A2/A4 secondary PCR products (10 µl) using ApoI (New England Biolabs Inc., Ipswich, Massachusetts, USA) restriction enzyme for 15 min at 50 °C following manufacturer’s instructions. The resulting DNA fragments were separated and resolved by gel electrophoresis on a 2% agarose gel stained with SYBR green at 100 V for 45 min. ApoI digests *Pfmdr1*PCR product when *Pfmdr1*N86 (wild type allele) is present. The PCR amplification was performed three consecutive times for a given sample in order to get a successful amplification. Also, the nested PCR products were subjected to RFLP using ApoI twice (with independent PCR products) to reconfirm the *Pfmdr1* N86Y alleles. Additionally, few random samples were chosen and were subjected to sanger sequencing.

### Data analyses

Chi square and Fisher exact tests were applied to compare the proportions of *Pfmdr1* N86Y alleles in this study. The statistical significance was set at *p*-value< 0.05.

### Ethical considerations

This study was approved by the Institutional Ethics Committee of Fondation Congolaise pour la Recherche Médicale, FCRM, Brazzaville, Republic of Congo. Written informed consent was obtained from all participants before samples collection. The objectives of the study including the study procedures, sample to be taken, study benefits, potential risks and discomforts were explained. Newly opened needle and syringe were used for each subject.

## Results

**Fig. 1:**
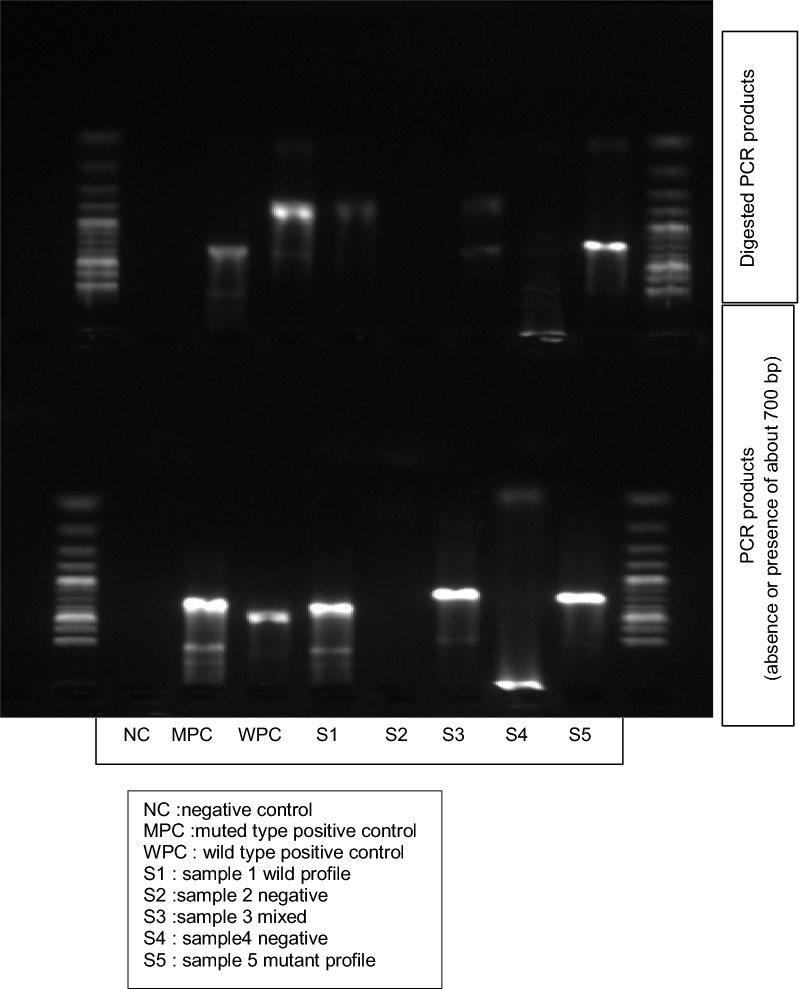
Electrophoresis gel before and after digestion by enzymes of restriction

The baseline characteristics of participants recruited in this study is summarized in Table 1. The mean age of participants was 23.7 ± 5.75 years. Overall, 24% of the pregnant women did not take intermittent preventive treatment during pregnancy and most of the participants (70%) had > 1 parity. Of the 101 matched samples analysed in this study, *Pfmdr1* was successfully amplified in 59 (58%), 38 (38%) and 21 (21%) maternal peripheral blood, placental blood and cord blood samples, respectively. Figure 1 shows an electrophoresis gel of PCR products before and after digestion with specific enzyme restriction. *Pfmsp2* genotyping showed mean multiplicity of infection (MOI) was 1.06 ± 0.24. High prevalence of *Pfmdr1* wild type allele (N86) was observed among the different sample types. *Pfmdr1* N86 was present in 70% (41/59), 58% (22/38) and 86% (18/21) of the peripheral blood, placenta blood and cord blood samples, respectively. The remaining samples had the mutant allele (*Pfmdr1*86Y). Three peripheral blood samples, two placental blood samples and one cord blood sample had both the wild type and mutant *Pfmdr1* N86Y alleles. The *Pfmdr1* N86 allele prevalence was similar (*p*-value> 0.05) among different sample types, parity and the number of intermittent preventive treatment during pregnancy (Table 2).

**Table 1:** Baseline characteristics of pregnant women with asymptomatic malaria

	No. of participants (n = 101)	Percentage (%)
Age group (years)		
15–30	89	88
> 30	12	12
Dose of IPTp		
0	24	24
1	28	28
≥ 2	49	49
Parity		
Primiparae	30	30
Secondiparae	33	33
Multiparae	38	38

IPTp, Intermittent preventive treatment in pregnancy

**Table 2:** Distribution of the *Pfmdr1*N86 allele among peripheral blood, placenta blood and cord blood samples from the Republic of Congo

	Peripheral blood n (%)	Placental blood n (%)	Cord blood n (%)
Dose IPTp			
0	8/14 (57)	6/9 (67)	5/6 (83)
1	13/18 (72)	4/13 (33)	8/10 (80)
> 2	20/27 (74)	12/16 (71)	5/5 (100)
*p*-value	0.512	0.251	0.569
Parity			
Primiparae	13/18 (72)	4/10 (40)	7/9 (78)
Secondiparae	12/18 (67)	10/10 (100)	6/6 (100)
Multiparae	16/23 (70)	8/18 (44)	5/6 (83)
*p*-value	0.936	0.007	0.475

IPTp, Intermittent preventive treatment in pregnancy

## Discussion

Anti-malarial drug resistance is a major obstacle in malaria reduction/eradication globally. Molecular surveillance is important to identify resistant phenotypes and to constantly monitor for any anti-malarial drug resistance. This study was set out to determine the prevalence of *Pfmdr1* N86Y mutation among pregnant women having a normal child delivery at the Madibou Integrated Health Centre, Brazzaville Republic of Congo. The *Pfmsp2* gene used to determine MOI showed mean multiplicity of infection as 1.06 ± 0.24. In areas with constant transmission of malaria, MOI may increase as immunity develops. MOI in pregnant women is a factor for the acquisition and maintenance of immunity against malaria. In this study, only using *msp2* genotyping, only one set of parasite clones were predominantly present among the pregnant women investigated. However, there are possibilities that these individuals may harbour more than one parasite, and this could be explained only by additional *msp1* genotyping for K1, MAD20, and RO33 alleles.

The frequency of *Pfmdr1* 86Y (mutated allele) in this study was lower than previously estimated in this setting in 2010 (73%) and in 2015 (27%) [27, 28]. These findings are also comparable to *Pfmdr1* 86Y allele (23%) global frequency and most parts of Africa (17 to 24%), except Central Africa, where high resistant allele frequency (44%) has been observed [29]. In Southeast Asia, however, the frequency of *Pfmdr1* 86Y is much lower than observed in this study whereas it has almost reached fixation in Papua New Guinea [29].

*Pfmdr1* N86Y mutation is known to modulate *P. falciparum* susceptibility to various anti-malarial drugs by regulating the influx of the drugs into the parasite’s digestive vacuole. Previous studies have shown that parasite carrying this mutation are less susceptible to 4-aminoquinolines, namely chloroquine, amodiaquine and piperaquine, in vitro [30] and increase the risk of chloroquine or amodiaquine therapeutic failure [15]. On the other hand, the *Pfmdr1* 86Y mutation enhance malaria parasite susceptibility to lumefantrine, mefloquine and the active derivative of artemisinin, dihydroartemisinin [30]. The converse impact of *Pfmdr1* N86Y on *P. falciparum* response to longer-acting partner drugs of ACT implies that wide spread use of AL and ASAQ, particularly in Africa, exert opposite selection pressure on *P. falciparum* populations and allele frequency [31, 32].

Changes in malaria treatment policies greatly influence the frequency of mutations that modulate *P. falciparum* susceptibility to anti-malarial drugs, including *Pfmdr1* N86Y [31]. The introduction of ACT in the early 2000s and cessation of chloroquine use in the 1990s led to drastic changes in *Pfmdr1* N86Y allele frequency in various malaria-endemic settings [27, 33, 34]. For instance, the frequency of *Pfmdr1*86Y has declined dramatically, in favour of *Pfmdr1* N86, in countries, where AL is used as the first − line treatment for malaria. The increase in *Pfmdr1* N86 allele frequency is faster when AL is used compared to ASAQ usage [31]. In areas where ASAQ is the primary treatment for malaria, the decline of *Pfmdr1* 86Y allele frequency is slow owing to the reduced susceptibility of parasites carrying this mutation to amodiaquine.

Previous studies demonstrate that parasites carrying *Pfmdr1* N86 tolerate higher lumefantrine levels and have short-time to reinfection or recrudescence in patients with high lumefantrine concentration following AL treatment [16, 17]. Even though there is no evidence directly linking *Pfmdr1* N86 to AL treatment failure and AL is still highly efficacious, parasite tolerance to lumefantrine is a clear warning sign for plausible emergence of resistance against AL. In this context, *Pfmdr1* 86 N can be used to track lumefantrine selective pressure in a given area [17]. The findings show that *Pfmdr1*N 86 allele is approaching fixation in the Republic of Congo and could provide the genetic background needed for the emergence of resistance against lumefantrine threatening AL usefulness in this setting. However, this possibility could be averted by concurrent use of AL and ASAQ as first-line treatment for uncomplicated *P. falciparum* malaria. Such a strategy is supported by evidence showing the opposite effect of both *Pfmdr1* N86Y alleles on *P. falciparum* susceptibility to AL and ASAQ [18].

## Conclusion

This study offers an update on the frequency of *Pfmdr1* N86Y alleles in Brazzaville, Republic of Congo and provides evidence supporting the concomitant deployment or rotation of AL and ASAQ as the primary treatment for uncomplicated *P. falciparum* malaria. This will be helpful to halt any further selection of *Pfmdr1* alleles that dampen parasite susceptibility and safeguard AL efficacy.

## Data Availability

All raw data provided in this work are available upon request to the corresponding author.

## Abbreviations

ACT: Artemisinin-based combined therapy
AL: Artemether–lumefantrine
ASAQ: Artesunate–amodiaquine
CQ: Chloroquine
FCRM: Fondation Congolaise pour la Recherche Médicale
IPTp: Intermittent preventive treatment for pregnant women
IPTi: Intermittent preventive treatment for infants
SNP: Single nucleotide polymorphism
SP: Sulfadoxine-pyrimethamine

## References

[1] WHO (2015). Guidelines for the treatment of malaria.

[2] Trape JF (2001). The public health impact of chloroquine resistance in Africa. Am J Trop Med Hyg.

[3] Conrad MD, Rosenthal PJ (2019). Antimalarial drug resistance in Africa: the calm before the storm?. Lancet Infect Dis..

[4] Hamilton WL, Amato R, van der Pluijm RW, Jacob CG, Quang HH, Thuy-Nhien NT (2019). Evolution and expansion of multidrug-resistant malaria in southeast Asia: a genomic epidemiology study. Lancet Infect Dis..

[5] Ariey F, Witkowski B, Amaratunga C, Beghain J, Langlois AC, Khim N (2014). A molecular marker of artemisinin-resistant *Plasmodium falciparum* malaria. Nature.

[6] Ashley EA, Dhorda M, Fairhurst RM, Amaratunga C, Lim P, Suon S (2014). Spread of artemisinin resistance in *Plasmodium falciparum* malaria. N Engl J Med.

[7] Mita T, Tanabe K, Kita K (2009). Spread and evolution of *Plasmodium falciparum* drug resistance. Parasitol Int.

[8] Nsimba B, Malonga DA, Mouata AM, Louya F, Kiori J, Malanda M (2004). Efficacy of sulfadoxine/pyrimethamine in the treatment of uncomplicated *Plasmodium falciparum* malaria in Republic of Congo. Am J Trop Med Hyg.

[9] Mayengue PI, Ndounga M, Davy MM, Tandou N, Ntoumi F (2005). In vivo chloroquine resistance and prevalence of the pfcrt codon 76 mutation in *Plasmodium falciparum* isolates from the Republic of Congo. Acta Trop.

[10] Ndounga M, Tahar R, Basco LK, Casimiro PN, Malonga DA, Ntoumi F (2007). Therapeutic efficacy of sulfadoxine-pyrimethamine and the prevalence of molecular markers of resistance in under 5-year olds in Brazzaville, Congo. Trop Med Int Health..

[11] Koukouikila-Koussounda F, Bakoua D, Fesser A, Nkombo M, Vouvoungui C, Ntoumi F (2015). High prevalence of sulphadoxine-pyrimethamine resistance-associated mutations in *Plasmodium falciparum* field isolates from pregnant women in Brazzaville, Republic of Congo. Infect Genet Evol..

[12] Cowman AF, Karcz S, Galatis D, Culvenor JG (1991). A P-glycoprotein homologue of *Plasmodium falciparum* is localized on the digestive vacuole. J Cell Biol.

[13] Reiling SJ, Rohrbach P (2015). Monitoring PfMDR1 transport in *Plasmodium falciparum*. Malar J..

[14] Price RN, Uhlemann AC, Brockman A, McGready R, Ashley E, Phaipun L (2004). Mefloquine resistance in *Plasmodium falciparum* and increased pfmdr1 gene copy number. Lancet.

[15] Picot S, Olliaro P, de Monbrison F, Bienvenu AL, Price RN, Ringwald P (2009). A systematic review and meta-analysis of evidence for correlation between molecular markers of parasite resistance and treatment outcome in falciparum malaria. Malar J..

[16] Otienoburu SD, Maiga-Ascofare O, Schramm B, Jullien V, Jones JJ, Zolia YM (2016). Selection of *Plasmodium falciparum pfcrt* and *pfmdr1* polymorphisms after treatment with artesunate-amodiaquine fixed dose combination or artemether-lumefantrine in Liberia. Malar J..

[17] Sisowath C, Stromberg J, Mårtensson A, Msellem M, Obondo C, Bjorkman A (2005). In vivo selection of *Plasmodium falciparum pfmdr1* 86 N coding alleles by artemether-lumefantrine (Coartem). J Infect Dis.

[18] Sondo P, Derra K, Diallo Nakanabo S, Tarnagda Z, Kazienga A, Zampa O (2016). Artesunate-amodiaquine and artemether-lumefantrine therapies and selection of *Pfcrt* and *Pfmdr1* alleles in Nanoro, Burkina Faso. PLoS One..

[19] Malmberg M, Ferreira PE, Tarning J, Ursing J, Ngasala B, Bjorkman A (2013). *Plasmodium falciparum* drug resistance phenotype as assessed by patient antimalarial drug levels and its association with pfmdr1 polymorphisms. J Infect Dis.

[20] Taylor AR, Flegg JA, Holmes CC, Guérin PJ, Sibley CH, Conrad MD (2017). Artemether-lumefantrine and dihydroartemisinin-piperaquine exert inverse selective pressure on *Plasmodium falciparum* drug sensitivity-associated haplotypes in Uganda. Open Forum Infect Dis..

[21] Gil JP, Krishna S (2017). *Pfmdr1* (*Plasmodium falciparum* multidrug drug resistance gene 1): a pivotal factor in malaria resistance to artemisinin combination therapies. Expert Rev Anti Infect Ther..

[22] Trape JF, Peelman P, Morault-Peelman B (1985). Criteria for diagnosing clinical malaria among a semi-immune population exposed to intense and perennial transmission. Trans R Soc Trop Med Hyg.

[23] Trape JF, Zoulani A (1987). Malaria and urbanization in central Africa: the example of Brazzaville. Part II: results of entomological surveys and epidemiological analysis. Trans R Soc Trop Med Hyg..

[24] Ministère de la Santé et de la Population (2006). Politique nationale de lutte contre le paludisme.

[25] Gueye NSG, Ntoumi F, Vouvoungui C, Kobawila SC, Nkombo M, Mouanga AM (2018). *Plasmodium falciparum* merozoite protein-1 genetic diversity and multiplicity of infection in isolates from Congolese children consulting in a pediatric hospital in Brazzaville. Acta Trop.

[26] Mungthin M, Khositnithikul R, Sitthichot N, Suwandittakul N, Wattanaveeradej V, Ward SA (2010). Association between the pfmdr1 gene and in vitro artemether and lumefantrine sensitivity in Thai isolates of *Plasmodium falciparum*. Am J Trop Med Hyg.

[27] Koukouikila-Koussounda F, Jeyaraj S, Nguetse CN, Nkonganyi CN, Kokou KC, Etoka-Beka MK (2017). Molecular surveillance of *Plasmodium falciparum* drug resistance in the Republic of Congo: four and nine years after the introduction of artemisinin-based combination therapy. Malar J..

[28] ACT Partner Drug Molecular Surveyor. (https://www.wwarn.org/tracking-resistance/act-partner-drug-molecular-surveyor).

[29] *Plasmodium falciparum* Community Project. (www.malariagen.net/projects/p-falciparum-community-project).

[30] Veiga MI, Dhingra SK, Henrich PP, Straimer J, Gnadig N, Uhlemann AC (2016). Globally prevalent PfMDR1 mutations modulate *Plasmodium falciparum* susceptibility to artemisinin-based combination therapies. Nat Commun..

[31] Okell LC, Reiter LM, Ebbe LS, Baraka V, Bisanzio D, Watson OJ (2018). Emerging implications of policies on malaria treatment: genetic changes in the Pfmdr-1 gene affecting susceptibility to artemether-lumefantrine and artesunate-amodiaquine in Africa. BMJ Glob Health..

[32] Venkatesan M, Gadalla NB, Stepniewska K, Dahal P, Nsanzabana C, Moriera C (2014). Polymorphisms in *Plasmodium falciparum* chloroquine resistance transporter and multidrug resistance 1 genes: parasite risk factors that affect treatment outcomes for *P. falciparum* malaria after artemether-lumefantrine and artesunate-amodiaquine. Am J Trop Med Hyg..

[33] Hayward R, Saliba KJ, Kirk K (2005). Pfmdr1 mutations associated with chloroquine resistance incur a fitness cost in *Plasmodium falciparum*. Mol Microbiol.

[34] Moyeh MN, Njimoh DL, Evehe MS, Ali IM, Nji AM, Nkafu DN (2018). Effects of drug policy changes on evolution of molecular markers of *Plasmodium falciparum* resistance to chloroquine, amodiaquine, and sulphadoxine-pyrimethamine in the South West Region of Cameroon. Malar Res Treat..

